# Tensions as productive forces in coproduced mental health research: an explorative qualitative study

**DOI:** 10.1186/s40900-026-00934-0

**Published:** 2026-06-29

**Authors:** Anneli Gustafsson, Hilda Näslund, Urban Markström, Conny Allaskog, Filippa Gagnér Jenneteg, Elisabeth Argentzell, Katarina Grim, Petra Svedberg

**Affiliations:** 1 Department of Social Work, Umeå University, Umeå, Sweden; 2 The National Partnership for Mental Health, NSPH, Stockholm, Sweden; 3 The National Partnership for Mental Health in Västra Götaland and Gothenburg, Gothenburg, Sweden; 4 Department of Health Sciences, Lund University, Lund, Sweden; 5 Department of Social and Psychological Studies, Karlstad University, Karlstad, Sweden; 6 School of Health and Welfare, Halmstad University, Halmstad, Sweden

**Keywords:** Tensions, Coproduction, Mental health research, Service user involvement, Collaborative research practices, Tension management strategies, Power dynamics

## Abstract

**Background:**

In coproduced research, competing priorities among researchers and partners often surface as the work progresses, making such processes rarely tension-free. Although coproduction approaches are increasingly used in mental health research, there is limited understanding of how tensions are managed and how this influences collaboration. Hence, the aim of the study is to explore how tensions emerge within coproduced research by analysing the conditions, interactions, and processes influencing their emergence and management.

**Methods:**

This study was conducted within UserInvolve, a six‑year Swedish research programme involving a national service user organisation as its principal partner. Using an exploratory qualitative design, the study examined coproduction in the programme. Data were collected in two stages: (1) preparatory exploration through six group interviews concerning coproduction processes with subproject group participants, including researchers, representatives from service user organisations, national agencies, service providers (2) in-depth exploration through four group interviews focusing explicitly on tensions with a purposeful sample of participants from stage one. An abductive analytical approach was used, combining inductive interpretations with a deductive framework of four tension types: organising, learning, performance, and belonging. The analysis proceeded through four phases: exploratory analysis, condensation, collective interpretation and final synthesis within the author group.

**Results:**

All four tension types were identified, reflecting structural constraints, negotiations of knowledge, competing purposes and questions of legitimacy in collaboration. A finding was that dynamics of power — epistemic, organisational, and relational — cut across all tensions and shaped how participants interpreted situations, negotiated roles, and understood legitimate expertise. What appeared as practical challenges often reflected deeper shifts in authority, knowledge, and expectations. Tensions also acted as generative mechanisms that prompted participants to question assumptions and develop new ways of working. Strategies for managing tensions included adaptive reflexivity, shared learning, renegotiation of roles, and sustained relational investment.

**Conclusions:**

Tensions are inherent to coproduced mental health research and can function as productive forces that support and strengthen collaboration, learning, and epistemic development. Findings indicate that tension‑based approaches require greater attention to power, which fundamentally shapes how tensions arise and are managed. Recognising tensions as potentially generative may strengthen the design and evaluation of coproduced research.

**Supplementary Information:**

The online version contains supplementary material available at 10.1186/s40900-026-00934-0.

## Abstract (plain language summary)

Coproduced research involves people who use services, professionals, and researchers to work on shared projects. Because these groups have different roles and priorities, disagreements and practical pressures often appear as the work progresses. If these tensions are not handled well, coproduction may be undermined or break down. This study explores how tensions arise and are managed in coproduced mental health research by examining the conditions, interactions, and processes within UserInvolve, a large six-year Swedish research programme. A group of researchers and partners conducted a two-stage qualitative study to explore tensions. First, they performed a preparatory review. Then, they had in-depth group interviews with people involved in the programme about tensions. Then, they looked for patterns in how tensions arose and were handled over time. The group found that tensions were common and presented as disagreements about how the work was organised, what counts as knowledge, what the project is for and aiming to achieve and who is included and feels they belong. Across all of these factors, differences in power were central. Tensions were not only problems; when discussed openly, they helped teams question assumptions, adapt plans, and find better ways of working. Helpful responses included making time to reflect, learning together, agreeing and revisiting roles, and investing in relationships.

**Supplementary Information:**

The online version contains supplementary material available at 10.1186/s40900-026-00934-0.

## Background

Within the mental health research community, there is an increasing emphasis on different collaboration-based strategies [1–5], and this trend is further reflected in the growing expectation from research funders that research should be conducted through collaboration with civil society, service users (SU), patients and professionals [6]. A range of terms is used to describe collaboration and SU involvement in research, including coproduction, cocreation, codesign, peer research, user-focused research and Public Patient Involvement (PPI). Some approaches emphasise degrees of involvement, others foreground shared control, experiential knowledge and empowerment throughout the research process [7]. While acknowledging the existence of related and distinct concepts, coproduction is used throughout this paper because it best captures the form of collaboration studied, namely research conducted in partnership with mental health SUs, practitioners and other stakeholders, with an emphasis on shared knowledge generation aimed at enhancing the applicability and relevance of research in the mental health field [8]. Principles for successful coproduction often concern relationship building, the sharing of power, reciprocity, and the embracing of diverse perspectives and skills [9]. Even though coproduction can occur in research with individual users, it often implies a collective group process [10]. As such, coproduction research is interactive and dynamic [11, 12]. When research purposes, designs and data are understood through broadened expertise based on the integration of SUs and professional perspectives, researchers’ traditional identities as experts and as those in charge are modified [1].

In coproduced research, differing and sometimes competing values, demands and priorities among and between involved groups often surface as the process unfolds [13–17]. In other words, coproduction processes are seldom seamless or frictionless paths. Prior research indicates that the emergence of tensions and challenges can prompt a return of decision-making power to its traditional seat, with academic researchers reclaiming control over central methodological choices, a shift that effectively dilutes the “co” in co-research [18]. Since the management of coproduction research is expected to occur collaboratively between researchers and stakeholders, it is essential to examine how tensions emerge and are managed as well as what implications they have for coproduced research.

Despite the growing body of collaborative research in mental health research, there remains a limited in-depth understanding of how tensions emerge and are managed and how such management shapes the organisation of collaborative processes. In organisational and management research, the concept of tensions has gained substantial attention through paradox theory, where contradictory yet interrelated demands are conceptualised as persistent features of organising rather than as temporary problems to be resolved [19, 20]. Rather than framing challenges as obstacles to be removed, a tension perspective conceptualises them as inherent and enduring features of complex and dynamic systems. From this viewpoint, tensions are not only unavoidable but can also be productive, as they shape action, decision-making, and organisational development when actively engaged [19]. This perspective has also been introduced into innovation research in healthcare, where scholars argue that studies that focusing on tensions offers a more process-sensitive and generative foundation for understanding innovation than barrier-based approaches do [21–23]. More recently, tensions have been studied within coproduction in health research, where they have been shown to concern narratives of idealistic, tokenistic vs. realistic narratives, power and reciprocity, language and communication, and the relationship between individual motivation and structural conditions [17]. Taken together, this literature moves the focus beyond identifying challenges towards understanding how coproduction practices are shaped, negotiated, and adapted in context.

Applying a tension perspective to coproduction in mental health research is particularly relevant, as coproduced research involves multiple stakeholders, such as researchers, mental health service (MHS) representatives, and SUs, who operate according to different logics, goals, and accountability structures. Like the healthcare innovation processes described by Haring et al. [21], coproduction unfolds in contexts marked by interdependence, uncertainty, and pluralistic demands, making tensions between perspectives, roles, and expectations inherent features of the process. These tensions could be closely linked to action and to the future trajectory of coproduction, shaping both immediate decisions and longer-term relationships and outcomes. While such tensions are embedded in complex systems, they may remain latent until activated by specific conditions, such as shifts in power relations, resource constraints, or the introduction of new ways of working in response to changing institutional or policy contexts [19]. In light of this, a closer examination of how tensions are enacted and navigated within coproduced mental health research is warranted. Such knowledge may support researchers and stakeholders in identifying tensions early, reflecting on how they are handled, and developing more workable practices for coproduced research. Thus, the aim of this study is to explore how tensions emerge within coproduced research by analysing the conditions, interactions, and processes that influence their emergence and management.

## Methods

### The setting

This study is situated within UserInvolve, a six-year research programme grounded in an approach inspired by “doing it while troubling it” [24, 25], in which coproduction practices are implemented, critically examined, and continuously refined over time [5]. The research programme was established to advance coproduced, cross-disciplinary, and practice-relevant research on SU involvement strategies at the individual, organisational and systemic levels in the Swedish mental health sector [1]. The objective of the UserInvolve research programme is to develop and evaluate methods for meaningful coproduction between researchers and representatives from mental health SU organisations and MHS. SU representatives refer to both elected representatives of SU organisations and employees working within these organisations. MHS representatives refer to representatives from organisations and agencies engaged in the provision of mental health services. The programme consists of several empirical research projects, each focusing on specific SU involvement approaches, such as peer support, user-focused monitoring, and system-level SU involvement methods, all grounded in coproduction methodologies, involving not only the research community but also MHS representatives, as well as representatives from SU organisations in various coproduction groups and thematic workshops. A key partner in the research programme is the Swedish Partnership for Mental Health (NSPH), the leading national and regional umbrella organisation for SU associations in the Swedish mental health field, representing more than 70 000 individual patients, SUs and family carers [26]. As one of the most influential actors in shaping the national agenda for SU involvement in psychiatry and social psychiatry, NSPH plays an indispensable role: its coordination capacities, long-standing legitimacy, and sector-wide networks make it possible to operationalise the forms of rigorous and structured coproduction that underpin UserInvolve.

In UserInvolve, coproduction is understood not as a predefined method, but as an ongoing and situated practice that unfolds across the research process. Collaboration is enacted through continuous interactions, negotiations, and decisions within the specific settings and relationships in which the research takes place, and is shaped by changing project phases, conditions, and participant roles [1, 5]. This understanding captures research conducted in coproduction with mental health SUs, practitioners, and other stakeholders, emphasising shared knowledge generation to enhance the applicability and relevance of research in the mental health field [8]. Within this broader programme, the present study contributes to UserInvolve’s overall objective of developing knowledge about how coproduced mental health research can be organised and conducted by examining the tensions that arise in practice, how they are managed, and what they imply for research conduct and outcomes.

### Study design

This study employs an exploratory qualitative design [27] via abductive thematic analysis [28] to examine the interactions and relational dynamics through which coproduction is enacted across UserInvolve projects and participants. The analysis moves iteratively between empirical material and theory, applying the theory of tensions [19, 21] as an analytical lens to explore how competing demands and expectations unfold in practice.

### Data collection and participants

Data collection comprised two stages: an initial preparatory exploration and a subsequent in-depth study. In the preparatory phase, while the UserInvolve evaluation toolkit [5] was used, group interviews were conducted with researchers and stakeholders involved in the programme’s coproduction efforts at both the programme and subproject levels. These interviews (*n* = 6) were recorded, transcribed verbatim, and analysed to inform the design of the in-depth study. Although tensions were not an explicit focus, participants reflected on their experiences of coproduction, providing early indications of potential areas of tension.

Building on these initial insights, a second stage was undertaken through group interviews (*n* = 4) with key stakeholders involved in UserInvolve’s coproduction processes. The participants were selected via purposeful sampling [29], allowing for the inclusion of individuals with in-depth experience in coproduced research within the programme. These interviews enabled more detailed narratives about the emergence and experience of tensions within the overall research programme as well as within specific subprojects. All authors were also participants in the coproduction process under study. Researching one’s own practice entails methodological and ethical challenges, including risks of reduced reflexivity, familiarity bias, and constraints on open expression when interviewing close collaborators. These challenges may be particularly salient in mental health research, where power relations and stigma can influence what is articulated [30]. To address this, interview groups were composed with attention to creating balanced and supportive discussion dynamics, and interviews emphasised confidentiality and voluntary participation. The group interview format further supported collective reflection among participants on how tensions had been managed and on the implications these tensions had for the ongoing process and outcomes of the coproduced research. The interview guide began with an open introductory question inviting participants to describe situations in which they had experienced tensions in the coproduction processes. This was followed by thematic sections focusing on learning, performing, organising, and belonging tensions, with questions addressing how these tensions emerged, how they were experienced, and how they were managed. Probing questions were asked, such as ‘Can you provide examples’, ‘What did these tensions look like’, ‘Can you describe that further’. The data from the second phase constitute the empirical material analysed in the results section. Table 1 provides an overview of the characteristics of the data collected and the participants included in the study.

**Table 1:** Characteristics of the data collected and included participants

Research phase	Involved from UserInvolve	Data type	Participants (*n*)	Participant description	When
Preparatory exploration	UserInvolve overall strategy group	UI evaluation toolkit: Process-oriented evaluation group interview (*n* = 1)	12	SU representative (*n* = 4), MHS representative (*n* = 5), researcher (*n* = 3)	Midpoint of programme
	UserInvolve subproject: User-focused monitoring	UI evaluation toolkit: Process-oriented evaluation group interview (*n* = 1)	8	SU representative (*n* = 4), MHS representative (*n* = 1), researcher (*n* = 3)	Midpoint of project
		UI evaluation toolkit: Impact-oriented evaluation group interview (*n* = 1)	8	SU representative (*n* = 5), researcher (*n* = 3)	Postproject
	UserInvolve subproject: Peer support (substudy 1)	UI evaluation toolkit: Process-oriented evaluation group interview (*n* = 1),	5	SU representative (*n* = 3), researcher (*n* = 2)	Midpoint of project
	UserInvolve subproject: Peer support (substudy 2)	UserInvolve evaluation toolkit: Process-oriented evaluation group interview (*n* = 1)	7	SU representative (*n* = 4), MHS representative (*n* = 1), researcher (*n* = 2)	Midpoint of project
	UserInvolve subproject: SU involvement at systemic level	UserInvolve evaluation toolkit: Process-oriented evaluation group interview (*n* = 1)	11	SU representative (*n* = 7), MHS representative (*n* = 1), researcher (*n* = 3)	Midpoint of project
In-depth study	Key representatives involved in UserInvolve’s coproductions	Interactive group interview	4	SU representative (*n* = 3), researcher (*n* = 1)	Late midpoint of programme
	UserInvolve’s program group (group 1)	Interactive group interview	5	SU representative (*n* = 2), researcher (*n* = 3).	Lage midpoint of programme
	UserInvolve’s program group (group 2)	Interactive group interview	4	SU representative (*n* = 1), researcher (*n* = 3).	Late midpoint of programme
	Key MHS stakeholders involved in UserInvolve’s coproductions	Interactive group interview	5	MHS representative (*n* = 3), researcher (*n* = 2)	Late midpoint of programme

### Analytical framework

This study adopts an analytical approach drawing on the ideal types of tensions originally proposed by the organisation theorists Lewis and Smith [19]. In this study, tensions are understood as contradictory yet interrelated demands, goals, values, roles, or practices that coexist in coproduced research. Rather than being treated as temporary problems or barriers, tensions are analysed as persistent and dynamic features of collaboration that require ongoing navigation and management. The framework is well established in organisation and management research and has informed a growing interdisciplinary body of theoretical and empirical work [31]. The theoretical framework has also been established within the field of health innovation research [21, 22], which shares important features with research on coproduction in mental health. The decision to use an established analytical framework was informed by its high degree of recognisability among both researchers and partners within UserInvolve, which gave rise to rich discussions about tensions in coproduction in research as well as in collaboration processes in general. Further, it enables examination of how theoretically grounded tensions manifest in a new empirical context, in this case coproduction in mental health research. The framework builds on four ideal types of tensions: *organising*,* learning*,* performing* and *belonging tensions* [19, 21]. *Organising tensions* concern competing processes or organisational structures that meet during collaboration. *Learning tensions* arise when established practices are challenged through the introduction of new approaches, perspectives, or ways of working, often involving the renewal or dismantling of existing routines. *Performing tensions* emerge when stakeholders pursue differing or competing goals, creating tensions between cooperation and conflict due to the variety of goals and requirements of different groups engaging in collaboration. Finally, *belonging tensions* relate to clashes between stakeholders’ beliefs, values and identities. These ideal types are analytically distinct but often interrelated, with tensions emerging both within and across categories [19, 21]. Beyond the categorisation of tensions, tension theory explicitly foregrounds the management of tensions as a central analytical concern. This work draws on three management strategies for addressing tensions that have been identified: either/or, both/and, and more‑than approaches [19, 21, 32]. Either/or strategies address contradictory demands by privileging one element over another or by separating tensions across time or contexts. Both/and approaches seek to balance and integrate opposing elements simultaneously, recognising their interdependence. More‑than approaches aim to transcend existing tensions by developing new perspectives or solutions that reframe or move beyond the original alternatives.

### Data analysis

An abductive analytical approach was adopted, involving iterative movement between inductive and deductive reasoning across analytical phases. This interplay between data collection and analysis allowed emerging field insights to inform analytical focus and conceptual development. In line with the project’s coproducing orientation, the data analysis and the writing of this paper were jointly undertaken by a mixed author group bringing together different experiences and perspectives from the UserInvolve coproductions into the interpretive process. The author group comprised of researchers holding leading roles within the UserInvolve programme (UM, HN, EA, KG, PS); the UserInvolve coordinator, employed by NSPH to facilitate interaction between researchers and partners, and additionally appointed as a postdoctoral fellow two years into the program (AG); the chair of NSPH (CA); and a representative from a regional NSPH organisation involved in several of the programme’s projects (FGJ). Despite the complexity of AG’s combined role, she is referred to in the data as one of the SU representatives as this was the role she held throughout the programme and the primary position from which she reflected during the interviews. As already stated, researchers and coproduction partners are not only part of the empirical material under analysis—with their experiences, interactions, and reflections constituting key data—but also actively involved in interpreting and analysing this material cf. [24, 25]. To strengthen reflexivity in relation to this complexity, one author (PS), a researcher affiliated with the programme but not operationally involved in coproduction, contributed an external perspective. Reflexivity was further supported through ongoing discussions within the author group, and since it included both researchers (some with lived experience) and SU representatives, it enabled multiple perspectives on the data. The analysis was also designed as a systematic and iterative process, involving preliminary analysis, structured coding, analytical workshops, collective validation of emerging interpretations, and member-checking with participants. These procedures enabled the author group to critically examine assumptions, negotiate interpretations, and refine the analytical framing throughout the research process. While the authors’ close involvement may have shaped both the empirical material and the analytical interpretations, this positioning also provided in‑depth contextual insight into the coproduction process under study.

The systematic and iterative process analysis proceeded in four phases:

#### Preparatory exploration phase

AG, HN and PS conducted a preliminary analysis of the interview data from the preparatory exploration. On the basis of this initial exploratory work, the following research questions were formulated to guide the subsequent inquiry:

- What types of tensions arise during coproduction in research, and how are they experienced by participants?
- Under what conditions and through which interactions and processes do these tensions emerge?
- What strategies do participants use to cope with or manage these tensions?

Following this, the empirical material was provisionally categorised through the theoretical lens of organising, learning, performing or belonging tensions. The findings were discussed within the author group and shaped the design of the in-depth study.

#### Condensation of in-depth study

AG and PS condensed descriptions and variations into an analytical schema mapping tension and management strategies. A deductive component was added as AG coded material via the analytical framework categories, integrating illustrative quotations. This schema formed the basis for drafting the results section.

#### Collective analysis and validation

This step comprised four analytical workshops conducted between June 2025 and February 2026 with the author group. Interview transcripts and emerging interpretations were read and discussed collectively during the workshops, with a focus on identifying, refining, and negotiating interpretations of tensions in coproduction. These discussions informed iterative analytical decisions regarding coding, categorisation, and the use of the analytical framework. Between workshops, AG and PS further developed the coding and drafted the manuscript in line with the collective decisions made. As the analysis progressed, draft versions of the manuscript were read and discussed by the full author group, allowing for collective validation and refinement of the analytical framing.

**Finally**, member-checking [33] was conducted by sharing the results with all participants involved in the data collection, allowing them to review and comment on the interpretations to ensure that the findings accurately reflected their experiences. Before finalising the paper, the full author group critically reviewed it during a fifth workshop.

Together, these four phases structured the analytical process and ensured that interpretations were iteratively refined through both individual and collective work within the coproductive author group. The reporting followed and is described in the GRIPP2 Short Form [34], an internationally recognised, evidence-based and consensus-informed framework for documenting patient and public involvement in research (Supplementary Material 1). Language editing assistance was provided by Microsoft Copilot (M365 Copilot), which is logged in through Umeå University, to improve clarity, grammar and style. The authors reviewed and took full responsibility for the final content.

## Results

The findings are structured around the four identified tension types – *organising*,* learning*,* performance and belonging* – as well as the strategies participants used to navigate them. The empirical material is presented through subthemes illustrating how specific situations activate tensions between competing demands in coproduction (see Table 2).

**Table 2:** Analytical structure of tensions and strategies

Themes	Organising tensions	Learning tensions	Performing tension	Belonging tensions
***Tensions***				
Subthemes	Between rigid systems and lacking administrative recourses	Between knowledge ownership and interpretive authority	Between scientific neutrality and interest-based commitments	Between democratic representation and scientific standards
	Between hierarchy and equality	Between dependence and control	Between Time Efficiency and Meaningful Discussion	Between engagement and mandate
	Between momentum and long-term research timelines			
***Tensions management strategies***				
Themes	Adaptive reflexivity	Shared learning	Power renegotiation	Relational investment

### Organising tensions in coproduced mental health research

Organising tensions captures how participants face differing institutional logics, administrative capacities, and governance structures in coproduced mental health research. These tensions arise from mismatches between formal regulations, organisational resources, and expectations of equality and shared responsibility in coproduction. Rather than being tied to individual actors, organising tensions emerge at the intersection of institutional constraints and everyday collaborative practices, shaping how coproduction can be enacted in practice.

#### Between rigid systems and a lack of administrative resources

Researchers and SU representatives described how mandatory administrative and legal procedures within universities often slowed down or constrained collaborative work, particularly when these procedures were difficult to align with the organisational realities of SU organisations. Differences in organisational size, stability, and administrative capacity created unequal conditions for participation, shaping how collaboration could be practically organised. As one researcher described,

We can’t transfer money to them [an SU association] in the same way we do with other universities, because they don’t have any financial administrator resources… They would prefer to be employed by us, but then LAS [the Swedish Employment Protection Act] comes into play again, so we can’t hire them (1).

The participants highlighted how financial administration and employment arrangements were especially challenging across organisational boundaries. While universities operate within rigid systems governing payments, contracts, and labour law, many SU organisations lack comparable administrative infrastructure, relying instead on voluntary boards or external accounting support. These asymmetries generated tensions when standard academic procedures proved incompatible with SU organisations’ capacities or long-term sustainability, particularly when legal requirements implied permanent employment arrangements that were perceived as unfeasible within time-limited research programmes. The following quote from an SU representative serves as an illustration of this discussion:

The likelihood that academia would assume such permanent positions in programmes of this kind is extremely low (2).

These dynamics revealed underlying power imbalances in coproduction. Although coproduction is grounded in ideals of equality and shared responsibility, universities were described as benefiting from greater organisational resources and institutional stability. Moreover, if an SU representative is employed within academia, some degree of autonomy may also be lost, as one researcher reflected:

But it’s also a question that might be important if we want to keep some tension in coproduction. Perhaps we shouldn’t have everyone employed by the university; for example, if someone comes in to contribute a SU perspective and wants to stir things up a bit, that might be harder to do if they are also paid by the university (3).

#### Between hierarchy and equality

A central source of organising tension in the collaboration stemmed from the encounter between two markedly different organisational cultures, each with its own hierarchies. On the one hand, the academic environment was described as grounded in principles of academic freedom and individual scholarly independence. This produced a form of governance where decision-making was distributed, sometimes diffuse, and tied to disciplinary norms rather than organisational roles. As one SU representative explained, it was through dialogue that they realised:

[T]hese researchers have a sense of academic freedom and independence. You see the world a little differently there and have a different hierarchy than what we might have within our organisations (4).

Such reflections highlight how expectations about leadership, responsibility, and decision-making may clash when partners operate according to different institutional logics.

On the other hand, participants from the SU movement described themselves as working in highly operational contexts, carrying substantial responsibility and often possessing extensive professional and experiential expertise. However, these competencies did not necessarily translate into equivalent status within the wider academic system. As one SU representative articulated:

On the SU movement side, people may work in a very operational way, carry enormous responsibility, be highly educated, and have extensive experience. However, within the academic system, you are still essentially considered a junior lecturer (2).

#### Between momentum and long-term research timelines

Organising tensions also emerged around time horizons and resource allocation, reflecting differing organisational logics among coproducing participants. Time was frequently described as a central tension, with the rapid adaptability required in everyday practice contrasting with the slower, methodical pace of academic research. SU and MHS representatives stressed the need to respond quickly to shifting conditions, whereas researchers operated within predefined timelines shaped by funding structures and methodological requirements. These divergent temporal expectations were experienced as frustrating when research delays risk undermined momentum or continuity in practice settings, as this MHS representative reflected:

With respect to time and planning, we need to adapt quickly in the service. Research, on the other hand, works with a much longer perspective. By comparison, I think that SU organisations adjust every day to each situation (5).

Such temporal tensions were often compounded by constraints within the projects themselves. Research project leaders are typically allocated only a limited proportion of their working time—often approximately 20 per cent—to manage coproduced projects, even when multiple subprojects and many stakeholders are involved. From the perspective of SU representatives, this allocation appeared disproportionately small given the complexity and relational demands of coproduction, leading to frustration when expectations of leadership, coordination, and availability clashed with the realities of limited resources. As one SU representative reflected,

For us, that is an absurdly small amount of money to lead such a large project involving several subprojects and an incredibly complex coproduction, with perhaps 50–60 practitioners and their managers. […] we have almost laughed at how crazy it is: ‘What do they truly think?’(2).

### Learning tensions in coproduced mental health research

Learning tensions capture how participants face competing understandings of knowledge, expertise, and methodological approaches embedded in coproduced mental health research. Learning tensions arise when different forms of knowledge — scientific, experiential, and practice-based — are brought together, generating questions of knowledge ownership, interpretation, dependency and control. These tensions can manifest both internally and collectively, embodied in the experiences of both researchers and SU and MHS representatives, as they navigate the challenge of aligning personal convictions, institutional norms, and collaborative ideals across diverse contexts.

#### Between knowledge ownership and interpretive authority

The SU representatives expressed fear of having limited control over how their input and contribution would be theorised or presented in publications. These concerns were shaped by previous experiences in which isolated empirical statements had been disproportionately amplified in relation to the overall dataset, potentially leading to a negative reputation for the NSPH. This is illustrated in a quote by an SU representative:

You are passionate about something, driven by something; there is a reason why we are SU representatives, and you bring your own experience with you and feel that you represent so many other voices as well. You truly want what I say to be taken care of and presented in the right way. However, I can only control the process up to this point. Because then the result ends with someone who will interpret it, fit it into a theoretical framework, and break it apart. And that loss of control has always felt quite uncomfortable to me (6).

Within this specific concern, the material showed that meaningful participation required SU representatives to place considerable trust in researchers’ interpretive practices. As two distinct knowledge sources meet—experiential and academic—both implicitly carry a sense of interpretive precedence, generating a tension around who ultimately is in control of interpretations. Even though the SU and MHS representatives described that they often had the opportunity to engage in analysis and writing workshops, the tension around knowledge ownership appeared to persist to some extent. Reading research manuscripts in detail can be demanding for anyone and particularly challenging for those without formal research training in the field or for those who do not have English as their first language. Moreover, it may not always be possible to fully anticipate the potential consequences of the final manuscript. Despite the coproduction approach, there was an unspoken assumption among the SU and MHS representatives that the researchers ultimately have the final say in deciding on what knowledge to prioritise, as this MHS representative reflects:

It became clear that the researchers, not overwhelmingly, still emphasised quite a lot: “It’s good to do a mapping of what participation looks like […]” Those of us working in practice were much more interested in what forms of collaboration and participation we have and what they have resulted in—the outcomes. In addition, I also felt that the SU organisations talked a lot about relationships […] (5).

Moderator: Who makes the decision then?

MHS: My impression is that it’s the researchers. However, maybe that’s not how it is (5).

#### Between collaborative dependence and control

Researchers described their work as grounded in systematic and rigorous approaches while emphasising forms of dependence that were intrinsic to the collaborative set-up. In several UserInvolve projects, the involvement methods were developed within the SU movement and implemented in psychiatric and social psychiatric contexts. This meant that the SU representatives were the primary originators and, together with MHS representatives, indispensable knowledge holders of the approaches being investigated. This positioned researchers as reliant on coproduction partners not only for contextual understanding but also for access to relevant networks and arenas in the field. This dependence was described as essential for conducting meaningful research, yet it also generated internal methodological tensions related to control. One of the researchers reflected:

We simply could not manage without you [referring to SU and MHS representatives]. We would never understand the context […], if we did not have someone who could decode this and explain: “Here are these groupings, and here are the others.” Therefore, we are entirely dependent on you in that regard. This is the case of “it takes two to tango” in this type of research (1).

The notion of “exit”, that is, leaving the collaboration, was raised by researchers as a hypothetical counterpoint to this dependence, showing the tension between reliance on each other’s distinct expertise and the autonomy associated with that expertise. Nevertheless, SU representatives framed exit as a nonviable strategy and as something that their umbrella organisation has become quite experienced in avoiding, as one SU representative noted:

We’re not at that point, and we should acknowledge that this was something we struggled with in the early days when the NSPH was formed. Back then, there were many “exit” actors—movements unwilling to accept any compromise—and that was a cornerstone of our culture, with [name of person from the SU movement] at the forefront. Therefore, as individual member organisations, we cannot keep making absolute demands such as “otherwise we leave!” because then collaboration simply does not work. You truly have to be able to compromise (7).

### Performance tensions in coproduced mental health research

The performance tensions capture the participants’ competing purposes and expectations embedded in coproduced MHS research. These tensions do not follow clear participant boundaries but emerge across roles and positions, shaping how researchers, SU and MHS representatives engage with coproduction over time. They arise between divergent purposes, such as reaching emancipation versus science/enlightenment, idealism versus pragmatism, or differing expectations of coproduction outcomes. Furthermore, they can manifest both internally and collectively, embodied in the experiences of researchers and SU and MHS representatives when meeting the challenge of aligning personal convictions, institutional norms, and collaborative ideals across diverse contexts.

#### Between neutrality and interest-based commitments

Researchers described navigating expectations of neutrality while collaborating with participants pursuing normative or interest-based aims. This tension was often internally felt, arising when commitments to coproduction intersected with disciplinary norms of autonomy and objectivity. Researchers described how such tensions shaped their work, particularly in studies of recovery practices informed by lived experience. They recalled early resistance from psychiatric professionals, and later, as they were commissioned by an SU organisation to evaluate implementation efforts, they sensed an implicit expectation of positive results:

This created a new dilemma: being employed by [name of SU organisation] while evaluating something we were, in a sense, expected to endorse, which we could do—but it raised questions about neutrality. I felt it was much harder to remain neutral in this project than in others (8).

The initial critique from psychiatric professionals suggested that research on recovery practices challenged the established professional hierarchies and treatment paradigms. The stakes became more pronounced when researchers were later employed by an SU organisation that, in contrast, was seeking broader implementation of these practices within psychiatry and social psychiatry.

SU representatives emphasised the importance of robust scientific evidence to strengthen their methods and their legitimacy in national mental health services. On the other hand, they expressed concern over negative findings undermining advocacy efforts. These tensions were further shaped by organisational considerations, including time and resource limitations. As one SU representative explained,

I carry responsibility for the collaborations we commit to and invest our resources in. I have a responsibility to ensure that these efforts hopefully lead us somewhere—that they generate value. You [referring to researcher] need to present the results that exist, of course. However, if the results had been directly negative for us [in terms of the collaboration or its consequences], then we would have needed to sit down within NSPH and ask ourselves: ‘Can we truly do a second round of UserInvolve?’ Can we keep investing resources in something that, strategically, might undermine our ability to implement our own methods? That said, there is still a gain in terms of learning—we do learn more about our own methods (4).

Researchers expressed concerns related to expectations about purpose and outcomes, which became particularly visible in time-consuming coproduction processes. Academic context often emphasises publication output as central to career progression, a demand that contrasts sharply with the slower, relational pace of coproduced research. Activities such as building relationships, utilising diverse forms of knowledge and managing joint decision-making were highlighted as essential among the participants, but this time-consuming work tended to leave less time for writing. One of the researchers reflected upon balancing these competing expectations:

[G]o ahead and publish 15 articles so you can become an associate professor” […]. Coproduction is very time-consuming, as shown in the project at the beginning. A considerable amount of time was spent building relationships and juggling these tensions (9).

#### Between time efficiency and meaningful discussion

Other concerns emerged around differing assumptions about partners’ availability and preferred forms of involvement. Researchers expressed concerns that SU representatives and other stakeholders would have limited time for extended meetings. However, both the SU and MHS representatives described their engagement in the project as a priority and expressed a desire for more time together. As one SU representative explained,

I think there was one researcher in the group who said, “No, we can only have one-and-a-half-hour meetings. Otherwise, we won’t get anyone to be involved in the project group.” However, I think it was the SU representatives who wanted more meetings. Or at least longer meetings. Because there was so much information sharing and so little space for dialogue (2).

In this project, the research collaboration also functioned as a forum for collective reflection on the research topic, enabling the sharing of experiences across different geographical sites. While this was considered important, it was also time-consuming and reduced the time for narrowly focused research discussions. This illustrates how differing expectations regarding time, meeting structures and the purpose of engagement can generate tension, particularly when efforts to protect partners’ time inadvertently constrain opportunities for meaningful dialogue and collective learning.

### Belonging tensions in coproduced mental health research

Belonging tensions refer to the conflicts that arise from participants’ competing identities and memberships and the legitimacy claims attached to them. These tensions do not follow clear actor boundaries but emerge across roles and organisational positions, shaping how researchers, SU and MHS representatives experience inclusion, authority, and recognition within the coproduction process. Belonging tensions emerge when different forms of legitimacy, such as scientific credibility, democratic representation, and professional expertise, are mobilised to justify participation, influence, and decision-making. In doing so, they render questions of who belongs, on what grounds, and with what mandate both visible and contested.

#### Between democratic representation and scientific standards

Tensions around legitimacy were raised as the participants felt they needed to justify their presence or mandate within the coproduced research. These tensions raised concerns around who belongs in the collaboration and on what grounds. The participants proposed different justificatory logics: experiential or professional expertise, democratic representation, and scientific standards. As these logics intersected, questions of belonging became tied to recognition, identity, and authority within the collaboration.

For SU representatives from the NSPH, legitimacy was closely linked to democratic mobilisation and collective representation, which are central to the identity of the Swedish mental health SU movement. Their participation was understood as grounded in a mandate derived from a national umbrella organisation. As one SU representative noted,

NSPH as an organisation […] has been compromising for almost 20 years to make this work. Strong diplomacy and a great deal of effort have gone into keeping this common ship afloat. Therefore, it cannot be the case that one single organisation within the NSPH is given a much greater mandate than the others are. Instead, we need to think: “What are the common issues?” […] We have a mandate on the basis of the collective. We are democratically anchored (4).

This emphasis on collective mandates based on aggregated knowledge highlights concerns over research practices privileging certain voices or organisations over others, which in turn could cause fragmentation within the SU movement. Researchers, on the other hand, approached legitimacy claims through scientific norms and by critically examining assumptions about representativeness and political participation. Although framed as part of rigorous research practice, such scrutiny sometimes clashes with legitimacy rooted in identity, collective struggle, and long-term organisational work.

#### Between open membership and established partnership

This subtheme captures how early-stage uncertainties in the coproduced research created tensions between membership and the decision‑making mandate. In the early stages of the programme, there were no shared structures for collective decision‑making. This exposed and intensified tensions regarding membership, representation, and authority over key decisions. As decision-making arrangements between the researchers and the NSPH were still taking shape, both sides had to learn and adapt.

As a result, several early decisions about membership — later recognised as central to the programme’s direction and integrity — were made neither with sufficient consultation or grounding within the partnership nor with anchoring in scientific reasoning. This contributed to tensions related to belonging. One of the researchers reflected upon this:

In retrospect, I realised that some of these tensions—particularly when they became most visible—were the result of decisions we made very early in the process. For example, at that time, I had not fully internalised the idea that we were in a partnership. This also meant, in a sense, that I had taken a position: I considered NSPH to be the most suitable partner from a principled organisational perspective—the best representative of people with lived experience—for planning our programme. That, I now think, entails certain commitments. We need to take responsibility for that partnership. If, instead, one acts independently—assembling one’s own panels of people with lived experience or doing things differently—that creates friction (1).

#### Between engagement and mandate

While SU representatives described stepping into coproduction while navigating the collective under the umbrella of many member organisations, experiences of loneliness and powerlessness were emphasised among MHS representatives. These representatives reflected on their belonging in coproduction processes, drawing on their experience from working to strengthen the influence of SUs within their respective organisations. These individuals can arguably be seen as less representative of the broader MHS and more as boundary-spanning SU participation “champions” — actors who promote the importance of SU involvement and collaborative practices. The conflict between engagement and mandate was described by one of the MHS representatives:

In the role I have, I’m kind of… it’s a bit of a squeeze position. I mean, I have contact with SU organisations […]. I work to enable SU influence, but I don’t have any decision-making mandate. That creates frustration (10).

MHS representatives also described internal tensions between listening, learning and contributing, particularly when SU representatives articulated strong collective positions grounded in lived experience and advocacy. This sometimes led to uncertainty about their own role and contribution to coproduction. At the same time, they noted that the limited representation of the wider MHS hindered opportunities for constructive friction and mutual learning across professional perspectives, reinforcing feelings of marginality among the “champion” MHS representatives. As one MHS representative reflected,

If you think about health care, social services, or the regional and municipal levels, there are also differences— what kind of education do you bring with you? What lens do you wear when you enter into something? From a theoretical perspective, therefore, there are quite different entry points (11).

### Managing tensions in coproduced mental health research

The findings identified multiple tensions — *organising*,* learning*,* performance* and *belonging* — in coproduced mental health research. Rather than being resolved once and for all, these tensions persisted throughout the research process. Participants managed them through adaptive practices that enabled collaboration to proceed despite uncertainty, competing demands, and shifting conditions. More specifically, the findings show how either/or responses were sometimes used temporarily to stabilise collaboration, for example by clarifying roles, organisation, and coordination. More commonly, participants engaged in both/and responses, maintaining structure while remaining open to revision, and upholding scientific requirements while recognising experience-based knowledge as meaningful. In some instances, practices such as renegotiating power relations and investing in trust and relational safety moved beyond balancing tensions, instead reconfiguring the conditions for collaboration. The next section presents four analytical subthemes that capture how participants managed these tensions across the coproduction process.

#### Adaptive reflexivity

A central strategy for managing tensions involved adaptive reflexivity, characterised by collective reflection on earlier decisions and a readiness to revise them when needed. The participants described how representation, selection criteria, and participation structures were revisited over time as tensions surfaced or new insights emerged. This included acknowledging when initial decisions had been insufficient or poorly aligned with the realities of coproduction. Such reflexive practices enabled researchers and partners to develop a more pragmatic orientation toward coproduction, recalibrating expectations and purposes in response to emerging risks and challenges rather than adhering rigidly to initial plans. One SU representative reflected on how this gradual realisation led to increased organisational structuring and closer coordination within NSPH:

It became clear that there needed to be more structure around UserInvolve. There needed to be an organisation around it […]. At first, from the NSPH’s side, it sounded more like “Why haven’t the researchers thought about this?” However, looking back, I think it was a growing into the role—from both sides. Participating in UserInvolve and realising what our whole organisation could mean for such a comprehensive research programme (2).

#### Shared learning

Managing tensions also involved shared learning processes through which researchers, SU and MHS representatives deepened their understanding of each other’s forms of expertise, research practices, and organisational contexts. Through sustained engagement, participants gained greater insight into research design considerations, coproduction methodologies, and the principles underpinning collaborative knowledge production. At the same time, they developed a clearer understanding of how institutional structures, governance mechanisms, and organisational constraints shaped each partner’s room to manoeuvre. This mutual learning supported more informed negotiation of tensions related to knowledge, roles, and expectations. As one SU representative reflected,

I think it is a mutual learning process. We’ve also learnt to be clearer about what we think and how we think, without expecting that our views should be applied immediately and without discussion. That is not where we want to end up either. However, at times, we were afraid of causing friction. The more you get to know each other and a relationship develops, the more secure you become in putting words to what you think and feel, instead of holding back (7).

#### Power renegotiation

One of the strategies used to manage tensions was connected to the ongoing renegotiation of power and epistemic authority. The participants described a willingness to move beyond established comfort zones and to question taken-for-granted assumptions about who holds decision-making authority or defines legitimate knowledge. Managing tensions in this sense involved openness to expanding perspectives, accepting uncertainty, and engaging in dialogue about roles and responsibilities. Although such renegotiations were often experienced as demanding, they were viewed as necessary for sustaining coproduction as a shared endeavour rather than a researcher-led process. One researcher reflected on how experience enabled more strategic and context-sensitive communication:

Now I know how to avoid many tensions. What should I say first? What should I say quickly? I present one thing to the SU representatives and another to the region [health care provider]). There are two different PowerPoints, two different ways of presenting the project. It’s two different “sales.” They can’t be exactly the same if you want people on board (8).

#### Relational investment

Managing tensions requires sustained relational work and trust-building. The participants emphasised the need for additional meetings and flexible constellations to create space for dialogue, reflection, and repair when tensions emerged. Over time, such investments strengthened trust and made future tensions easier to navigate, although it was also noted that *exit* remained a latent option if trust could not be maintained.

In addition to these explicit practices, the participants highlighted the substantial everyday organisational and emotional labour required to sustain relational stability during coproduction, as illustrated in the following exchange:

Researcher: I’m sitting here thinking about the term “pressure specialist” […]. This idea involves having to deal with academic housework, or information and communication, and thinking a few steps ahead. Or having your antennae stretched far beyond yourself, so you can sense when something is starting to wobble somewhere (12).

SU: And that makes me think that I’m not sure all researchers are suited for that kind of work (2).

These remarks highlight the specialised relational and emotional competences involved in coproduction — skills that might extend beyond conventional academic training. However, participants emphasised that when such relational work was sustained, it contributed to a sense of psychological safety that allowed for open disagreement. As one SU representative reflected,

Today, I think we have a very safe, open, and straightforward discussion. We don’t always end up in the same position, but we dare to give space to what we think and feel. That is the most important thing—that no one owns a question, without an open and honest dialogue (7).

## Discussion

### Principal findings

The findings highlight four interrelated tensions shaping coproduced mental health research. *Organising tensions* reflects how institutional structures, resources, and governance arrangements constrain aspirations of equality in collaboration. *Learning tensions* centre on negotiations over knowledge, interpretation, and ownership when experiential and scientific expertise intersect. *Performance tensions* capture competing purposes and commitments, as participants balance scientific ideals with normative and pragmatic aims. *Belonging tensions* concern legitimacy and mandates, raising questions about who is recognised as a credible participant and on what grounds. However, the results showed that these categories alone were insufficient to explain how tensions unfolded in practice. Across the material, power emerged as a cross-cutting dimension, shaping how tensions were interpreted, experienced, and negotiated. Our findings suggest that power in coproduced mental health research operates not only through formal roles and organisational structures, but also through epistemic and relational processes that shape whose knowledge is recognised, whose participation is legitimised, and who can influence the direction of the research. Although the original tension framework by Lewis and Smith [19] does not focus on power, the lack of consideration of power dynamics has proven particularly consequential in the context of coproduction in mental health, where power asymmetries are deeply embedded in professional hierarchies, SU involvement structures, and the stigma associated with mental health challenges and services [5, 30, 35]. This finding resonates with van der Graaf et al.’s study of coproduction in collaborative health research, where power differences and lack of reciprocity were identified as one of four central tensions shaping coproduction practices [17]. However, our study extends this insight by showing that power is not only a discrete tension, but a cross-cutting dimension that influences how organising, learning, performing, and belonging tensions emerge, are experienced, and are managed in coproduced mental health research. Future theoretical development may benefit from integrating the dynamics of power more explicitly into the tension-based frameworks of coproduction, particularly in mental health contexts. To deepen the understanding of how inequalities regarding voice and knowledge influence coproduction in mental health research, future work could also draw on research examining how power inequalities, epistemic injustice and discursive practices shape tensions in organisations [36–41].

The theoretical contribution of this study shows that a tension perspective can move analyses of coproduction beyond descriptions of challenges or barriers by examining how competing demands are negotiated in practice. Rather than treating coproduction as an ideal model based on principles such as power sharing, reciprocity, and shared knowledge production, the tension perspective makes it possible to examine how these principles are negotiated when they meet institutional structures, scientific norms, stakeholder mandates, and unequal organisational conditions. The ways in which participants navigated *organising*,* learning*,* performance*, and *belonging tensions* consistently pointed to underlying negotiations of epistemic authority and what counts as legitimate participation. Such negotiations can be understood through the lens of epistemic injustice, a framework that foregrounds how social and institutional hierarchies shape who is recognised as a credible knower and contributor [38]. In coproduced research settings, power relations are embedded in both formal structures and everyday interactions, subtly influencing whose knowledge is foregrounded and how meanings are constructed [13, 42]. These dynamics, although rarely articulated explicitly by participants, became visible in how participants interpreted their roles, defended particular forms of expertise, and adjusted their expectations of one another. In this sense, tensions do not merely arise in specific situations; they reveal the ongoing work of making space for diverse epistemic positions and renegotiating the terms of collaboration. While tensions in coproduction can be approached through structural adjustments in how collaboration is organised, our findings suggest that they also concern deeper epistemic questions of authority, recognition, and legitimacy cf. [13, 37, 43].

Importantly, the findings also show that tensions were actively managed through a set of interrelated strategies, including adaptive reflexivity, shared learning, renegotiation of power, and sustained relational investment. Rather than seeking to resolve tensions conclusively, participants worked with and through them over time, enabling collaboration to continue despite uncertainty and competing demands. In doing so, tensions became catalysts for reflection and learning, gradually reshaping relationships and collective understandings of what meaningful coproduction entails. Tensions are not only challenges but can also be productive, prompting participants to question assumptions, broadening their perspectives, and moving beyond familiar ways of thinking and working. From this perspective, movement toward coproduction is not a discrete event or agreement but rather a gradual epistemic and relational process in which participants collectively renegotiate what counts as valid knowledge, legitimate participation, and shared purpose. Tensions therefore function not as disruptions to collaboration but as essential drivers of change over time. These dynamics align with prior work emphasising the generative potential of productive tensions in coproduction cf. [17, 24, 44].

The findings have several practical implications for designing and facilitating coproduced mental health research. First, coproduction processes may benefit from the recognition of tensions as expected and potentially productive rather than as signs of failure. Second, project leaders and coordinators may benefit from explicitly addressing power dynamics — both epistemic and organisational — and creating structures that support safe disagreement, shared sensemaking, and iterative adjustments during the coproduction process cf. [45]. Third, relational work, emotional labour, and communication require intentional resourcing, as these practices underpin the management of tensions over time cf. [7]. Finally, training for coproducing researchers may need to incorporate skills related to reflexivity, negotiation, and collaborative leadership.

### Methodological considerations

In our study, we used the concepts of credibility, dependability, and transferability to address issues of trustworthiness in qualitative research. These criteria, proposed by Graneheim et al. [46], provide a well-established framework for evaluating the rigor and quality of qualitative research.

The analytical framework enabled us to identify and systematise the tensions and how they were managed, providing a structured lens for interpreting participants’ experiences. Although the broader framework includes nine tension types, five of which are hybrid and intermediate tensions, we focused on the four primary categories. Analysing all nine would likely have added conceptual complexity but without clear analytical benefit, particularly given our abductive approach and the empirical difficulty of assigning participants’ experiences to specific tension types. Moreover, as we examined both how tensions emerged and how they were managed, applying the full framework risked reducing rather than enhancing analytical clarity. This methodological choice supported dependability by ensuring clarity and consistency in the analysis.

Credibility was strengthened through the coproduction process with SU representatives, who contributed to shaping the analytical approach, refining interpretations, and challenging preliminary readings of the data. This dialogical engagement functioned as an ongoing form of interpretive validation. Credibility was also supported by the mixed composition of the author group, which included researchers, some with lived experience, and SU representatives, thereby enabling multiple perspectives on the data. The inclusion of SU representatives as co-authors is still relatively uncommon in research but was important in this study because it allowed experiential, professional, and research-based knowledge to inform the interpretation of the findings. At the same time, several authors’ close involvement in UserInvolve provided in-depth contextual understanding of the coproduction process, while also creating risks of familiarity bias, taken-for-granted assumptions, and researcher authority shaping the analytical interpretations. To address these potential limitations, emerging findings were discussed in repeated analytical workshops, and one author affiliated with the programme but not operationally involved in coproduction contributed a more external perspective. However, as only a representative sample of individuals involved in the UserInvolve programme was interviewed, some perspectives from both SU and MHS representatives are not represented. While this does not undermine the internal credibility of the findings, it may limit the breadth of experiential variation captured.

Finally, transferability is shaped by the contextual embeddedness of the study within the Swedish mental health and welfare system and the organisational cultures, histories, and power dynamics. Sweden’s strong tradition of social movements has influenced expectations around citizen engagement, collective action, and state–civil society relations [47, 48]. While the conceptual insights regarding tensions and power in coproduction may be analytically transferable, the specific ways in which tensions emerge and are managed may differ in other national settings or coproduction traditions.

## Conclusion

This study demonstrates that tensions can be understood as inherent to coproduced mental health research and operate not as barriers that should be avoided but rather as generative forces that shape and strengthen collaboration, learning, and epistemic change. By analysing both the emergence of tensions and the strategies used to manage them, the findings deepen the understanding of how coproduction in mental health research unfolds in practice and highlights the central role of power, reflexivity, and relational work. These insights provide a foundation for advancing both the theory and practice of coproduced research in mental health and beyond. Conversely, by examining tensions in the context of coproduction in mental health research, this study contributes to organisational and management research by extending its empirical scope to a previously underexplored field. Further research could deepen the theoretical understanding of how power operates within tension-based frameworks of coproduction and examine how different tensions and management strategies overlap and interact in the practice of coproduced mental health research.

## Supplementary Information

Below is the link to the electronic supplementary material.

Supplementary Material 1

## Data Availability

The datasets analysed in this study are not publicly available to protect participant confidentiality, but anonymised data may be obtained from the corresponding author upon reasonable request.

## Abbreviations

SU: Service user
MHS: Mental health service
NSPH: The Swedish Partnership for Mental Health (Nationell samverkan för psykisk hälsa)
