# Supplementary material for: Tensions as productive forces in coproduced mental health research: an explorative qualitative study
Source: Res Involv Engagem. 2026 Jun 29;12:103. doi: 10.1186/s40900-026-00934-0 (PMC13317245; doi:10.1186/s40900-026-00934-0)
Supplement: Supplementary file 1 — Supplementary Material 1 [file 40900_2026_934_MOESM1_ESM.docx]

Patient and public involvement in GRIPP2 reporting checklist

| **Section and topic** | **Item** |
| --- | --- |
| 1: Aim | **Report the aim of PPI in the study.** The aim of PPI in this study was to incorporate diverse perspectives from participants involved in coproduction activities within the UserInvolve programme, ensuring that these viewpoints informed the study’s analytical focus and interpretation. |
| 2: Methods | **Provide a clear description of the methods used for PPI in the study.** PPI was integrated through a coproducing author group involving SU‑representatives and researchers who jointly contributed to the design, data analysis, interpretation, and manuscript development. Additional workshops enabled collective reflection and iterative validation of emerging findings. |
| 3: Study results | **Outcomes – Report the results of PPI in the study, including both positive and negative outcomes.** PPI contributed to the refinement of analytical categories and supported interpretations that reflected diverse perspectives within the programme. A key challenge was the extensive volume of interview material, which meant that not all authors had the time or capacity to engage with the full dataset; consequently, a smaller subgroup conducted preliminary analyses and drafted initial manuscript sections. This highlights the practical difficulty of implementing fully comprehensive PPI across all analytical stages. |
| 4: Discussion and conclusions | **Outcomes–Comment on the extent to which PPI influenced the study overall. Describe positive and negative effects.** PPI had a substantial influence on the study by shaping the analytical framing, refining interpretations, and strengthening the overall credibility of the findings. However, the process required time and engagement from participants already heavily involved in UserInvolve research programme activities, which limited the extent to which all participants could be fully integrated in every phase of the analysis and reporting activities. |
| 5: Reflections/critical  perspectives | **Comment critically on the study, reflecting on the things that went well and those that did not, so others can learn from this experience.** Collaborative work and open dialogue across roles functioned well and supported shared interpretation. A challenge was the limited time available to partners, which made it difficult to involve a mental health services representative in the author group without placing additional demands on them. Instead, we used a member‑checking process, enabling these partners to validate the results in writing at the final stage. |
